# Normalized STEAM-based diffusion tensor imaging provides a robust assessment of muscle tears in football players: preliminary results of a new approach to evaluate muscle injuries

**DOI:** 10.1007/s00330-017-5218-9

**Published:** 2018-02-08

**Authors:** Chiara Giraudo, Stanislav Motyka, Michael Weber, Manuela Karner, Christoph Resinger, Thorsten Feiweier, Siegfried Trattnig, Wolfgang Bogner

**Affiliations:** 1 High Field MR Center, Department of Biomedical Imaging and Image-guided Therapy, Medical University of Vienna, Waehringer Guertel 18-20, 1090 Vienna, Austria; 2 Orthopedic Department, Evangelisches Krankenhaus Wien, Vienna, Austria; 3 Siemens Healthcare GmbH, Erlangen, Germany; 4 Christian Doppler Laboratory for Clinical Molecular MR Imaging, Medical University of Vienna, Vienna, Austria

**Keywords:** Diffusion tensor imaging, Magnetic resonance imaging, Muscle, Injury, Athletes

## Abstract

**Objectives:**

To assess acute muscle tears in professional football players by diffusion tensor imaging (DTI) and evaluate the impact of normalization of data.

**Methods:**

Eight football players with acute lower limb muscle tears were examined. DTI metrics of the injured muscle and corresponding healthy contralateral muscle and of ROIs drawn in muscle tears (ROI_tear_) in the corresponding healthy contralateral muscle (ROI_hc_t_) in a healthy area ipsilateral to the injury (ROI_hi_) and in a corresponding contralateral area (ROI_hc_i_) were compared. The same comparison was performed for ratios of the injured (ROI_tear_/ROI_hi_) and contralateral sides (ROI_hc_t_/ROI_hc_i_). ANOVA, Bonferroni-corrected post-hoc and Student’s t-tests were used.

**Results:**

Analyses of the entire muscle did not show any differences (*p*>0.05 each) except for axial diffusivity (AD; *p*=0.048). ROI_tear_ showed higher mean diffusivity (MD) and AD than ROI_hc_t_ (*p*<0.05). Fractional anisotropy (FA) was lower in ROI_tear_ than in ROI_hi_ and ROI_hc_t_ (*p*<0.05). Radial diffusivity (RD) was higher in ROI_tear_ than in any other ROI (*p*<0.05). Ratios revealed higher MD and RD and lower FA and reduced number and length of fibre tracts on the injured side (*p*<0.05 each).

**Conclusions:**

DTI allowed a robust assessment of muscle tears in athletes especially after normalization to healthy muscle tissue.

****Key Points**:**

• *STEAM-based DTI allows the investigation of muscle tears affecting professional football players*.

• *Fractional anisotropy and mean diffusivity differ between injured and healthy muscle areas.*

• *Only normalized data show differences of fibre tracking metrics in muscle tears.*

• *The normalization of DTI-metrics enables a more robust characterization of muscle tears.*

## Introduction

Acute muscle injuries are very common in elite and non-elite athletes, and tears due to indirect active traumatic events especially have a high prevalence [1, 2]. In the last decades, the clinical evaluation of muscle strains has increasingly been associated with imaging-based assessment [2, 3]. Several grading systems of muscle injuries have been proposed in the clinical and radiological literature [4–6] and recently the Munich Consensus Statement highly recommended the use of an accurate terminology about muscle lesions [7]. Nevertheless, the prevalent MRI-based classification is still based on a rough quantification of the amount of torn fibres [8] preventing a high imaging-based accuracy in both therapeutic and prognostic management of patients [9]. Diffusion tensor imaging (DTI) [10–12] has been successfully used to investigate muscle tears on an animal model (i.e. dystrophic and wild mice) [13] as well as in patients (i.e. two patients with acute muscle tears) [14]. Even though DTI allows an accurate assessment of muscle anatomy [15–18] and disorders [13, 14, 19–21], it is affected by challenges (i.e. short T2 relaxation times of muscle) [22] and artifacts [23–25]. The development of new techniques for muscle fibre-tracking is, therefore, an active field of research [26–28]. Promising results were recently obtained using a Stimulated Echo Acquisition Mode (STEAM) sequence, which, among other advantages, is hardly affected by eddy current distortions and enables long diffusion times without strong T2-induced signal-to-noise ratio (SNR) loss via the application of mixing time (TM) [25, 29].

Despite the above-mentioned encouraging results and technical improvements, to the best of our knowledge a prospective study applying STEAM-DTI for investigating acute muscle tears in athletes has not been performed yet. Therefore, the main aim of this study was to assess and quantify acute muscle tears affecting the lower limb of professional football players with STEAM-DTI. As it has been demonstrated that in athletes differences between the muscles of the preferred and non-preferred leg occur [30–33], the second aim of this study was to evaluate the impact of a normalization of the data by deriving a ratio between injured and healthy areas on the injured limb and healthy areas on the contralateral extremity.

## Materials and methods

### Patients and study design

Eight professional football players (all males, age range 20–36 years) with clinically diagnosed acute muscle tears (i.e. < 1 week) of the lower limb were enrolled in this prospective, IRB-approved study. Written informed consent was obtained from each patient.

### MR protocol

Each patient was investigated on a 3T MAGNETOM Trio, a Tim system MRI Scanner (Siemens Healthcare, Erlangen, Germany) using a combination of an anterior four-channel matrix coil and a 12-channel spine coil. Both limbs (i.e. the injured and the healthy contralateral) were covered by a single STEAM-DTI scan with the following parameters: repetition time/echo time/TM (TR/TE/TM) 6,100 ms/30 ms/186 ms, 128 × 96 matrix, field of view (FOV) 440 × 330 mm^2^, GeneRalized Autocalibrating Partial Parallel Acquisition-2 (GRAPPA-2), diffusion time 200 ms, fat saturation (frequency selective suppression and gradient reversal), b-values 0 and 500 s/mm^2^, six averages, 12 directions; 30 adjacent axial slices of 3.5-mm thickness, time of acquisition (TA) 8:10 min; voxel volume 3.4 × 3.4 × 3.5 mm^3^.

For the morphological assessment, only the injured limb was imaged via positioning-matched, axial (TR/TE 3,000 ms/26 ms, matrix 384 × 384, FOV 220 × 220mm^2^, TA=1:18 min), coronal and sagittal proton density fat-sat (TR/TE 4,600 ms/26 ms, matrix 384 × 384, FOV 400 × 400 mm^2^, TA=4:18min, each) and axial T1-weighted TSE (TR/TE 921 ms/11 ms, matrix 448 × 448, FOV 220 × 220 mm^2^, TA=4:23 min) with 3-mm slice thickness.

### Morphological assessment

Each injury was rated according to the Munich Consensus classification (i.e. minor partial, moderate partial and (sub)total muscle tear/tendinous avulsion) [7] by a musculoskeletal radiologist (C.G., 6 years of experience in musculoskeletal radiology) using all morphological datasets.

### DTI post-processing

DTI images with the same contrast were co-registered to correct gross motion artifacts and/or misalignment [25]. Since STEAM-DTI images are affected by random artifacts due to involuntary muscle contractions [23, 25], a recent correction method, based on the weighted mean of voxels’ signal intensity (WMSI), was applied [25]. Then, a second co-registration among images from the same slice but with different diffusion gradient directions was used [25].

Masking was performed by multiplying MD and RD maps [25]. Matlab (The Mathworks, Natick, MA, USA) was used for the artifact correction, for both co-registrations and masking.

A fourth-order Runge-Kutta (RK4) tracking algorithm (DSI Studio,http://dsi-studio.labsolver.org) (FA and angular threshold 0.12 and 17°, respectively) [25, 34] was applied.

### DTI quantitative evaluation

#### Entire muscle analyses

DTI metrics (i.e. fractional anisotropy (FA), mean (MD), radial (RD) and axial (AD) diffusivity, number, length and volume of fibre tracts) were collected, after manual segmentation, from the entire examined section of the injured muscle and from the healthy contralateral corresponding muscle using DSI Studio (i.e. using b0 and PD-FS images in the background as anatomical reference). The contralateral leg was chosen as control, rather than control participants, because of the high inter-subject variability in DTI measurements [35–38].

#### Region of interest (ROI) analyses

**Fig. 1:**
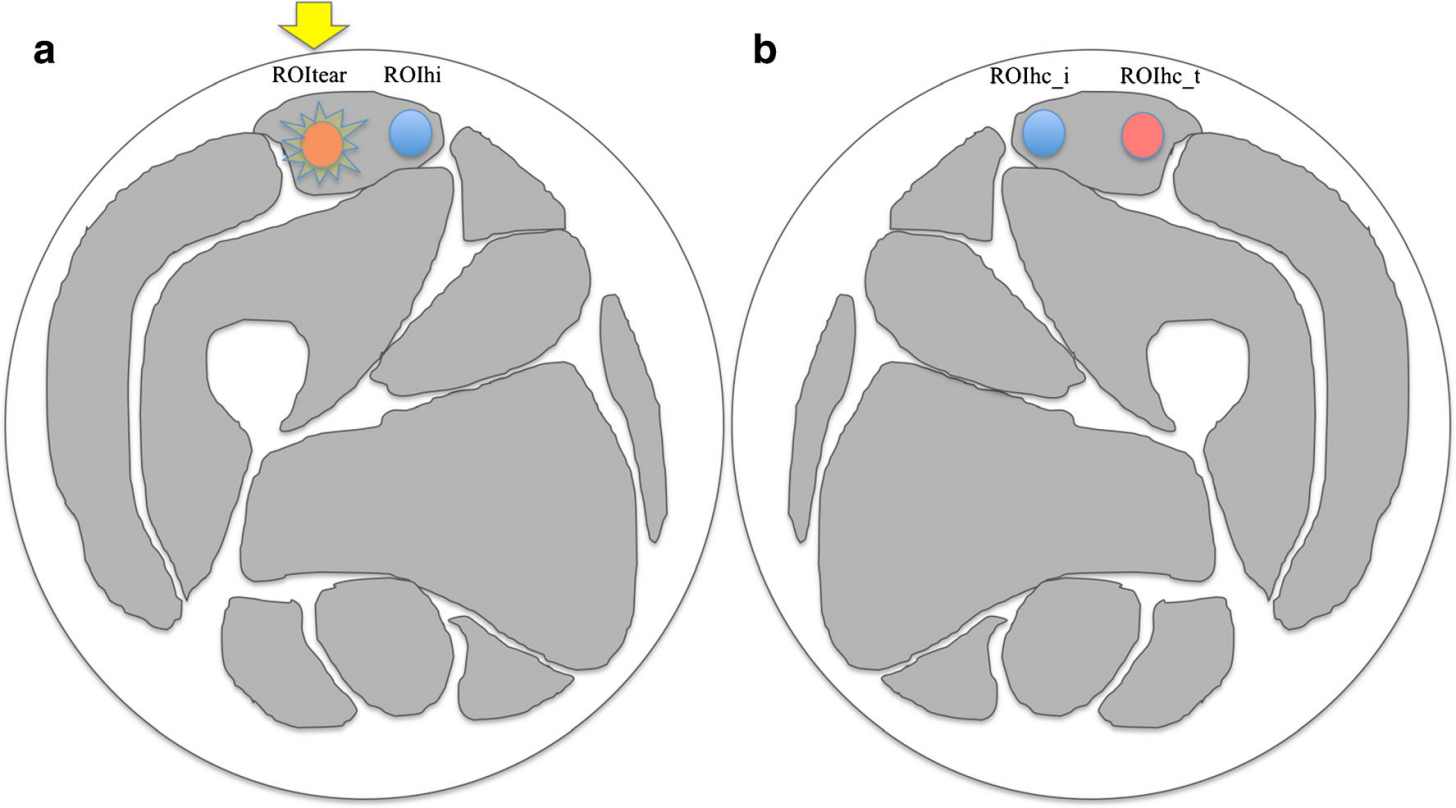
Drawing of the muscles of the thigh representing the regions of interest (ROIs) used for the ratio analysis. In this example, an injured area on the right rectus femoris muscle is represented (yellow star) where a manual ROI (red ROI in **a**, indicated by the yellow arrow) has been drawn (ROI_tear_). The same ROI has been drawn on a healthy ipsilateral area (blue ROI in **a**; i.e., ROI_hi_). The same areas were then investigated on the contralateral side (red and blue ROIs in **b**, respectively ROI_hc_t_ and ROI_hc_i_)

Freehand regions of interest (ROIs) were drawn along the margins of each muscle tear (ROI_tear_) (i.e. using b0 and PD-FS images in the background as anatomical reference) and the same ROI was applied on the corresponding healthy contralateral muscle (ROI_hc_t_). To rule out any physiological difference between right and left limbs, two other ROIs were drawn, both in healthy tissue: one in a healthy area ipsilateral to the injury (ROI_hi_) and one in a matching area in the contralateral limb (ROI_hc_i_) (Fig. 1).

#### Ratio

As it has already been demonstrated in the literature, differences between the muscles of the dominant and contralateral limb may occur in professional athletes [30–33]. Thus, to avoid any bias, an intra-subject normalization of DTI metrics was performed: ratios of DTI metrics of the injured side (ROI_tear_/ROI_hi_) and of the two corresponding contralateral healthy areas (ROI_hc_t_/ROI_hc_i_) were compared.

### Statistical analysis

Descriptive statistics were applied for categorical data. One-way repeated measures analysis of variance (ANOVA) with Greenhouse-Geisser correction and Bonferroni post-hoc tests were used to evaluate differences among all the examined ROIs. Student’s t-tests were applied to compare DTI metrics of the entire muscles as well as ratios of DTI metrics of the injured side (ROI_tear_/ROI_hi_) and of the corresponding contralateral healthy areas (ROI_hc_t_/ROI_hc_i_).

All statistical analyses were performed with SPSS Statistics 21.0 (IBM Corp, Armonk, NY, USA), and the level of significance was set at *p*<0.05.

## Results

Five out of the eight investigated patients showed an injury of the thigh and three one of the calf. Seven lesions affected the right side and one the left. Two tears were rated as minor partial and six as moderate [7] (Table 1).

**Table 1:** Demographic and clinical findings of the patients with muscle tears enrolled in the study

Gender		8 males
Age range		20–36 years
Injured muscle		
	Gastrocnemius medialis	2
	Rectus femoris	2
	Semimembranosus	1
	Semitendinosus	1
	Soleus	1
	Biceps femoris	1
Grading^#^	Minor partial tear	2
	Moderate partial	6
	(Sub)Total rupture	/

^**#**^According to the Munich Consensus’ classification

### DTI quantitative evaluation

#### Entire muscle

**Fig. 2:**
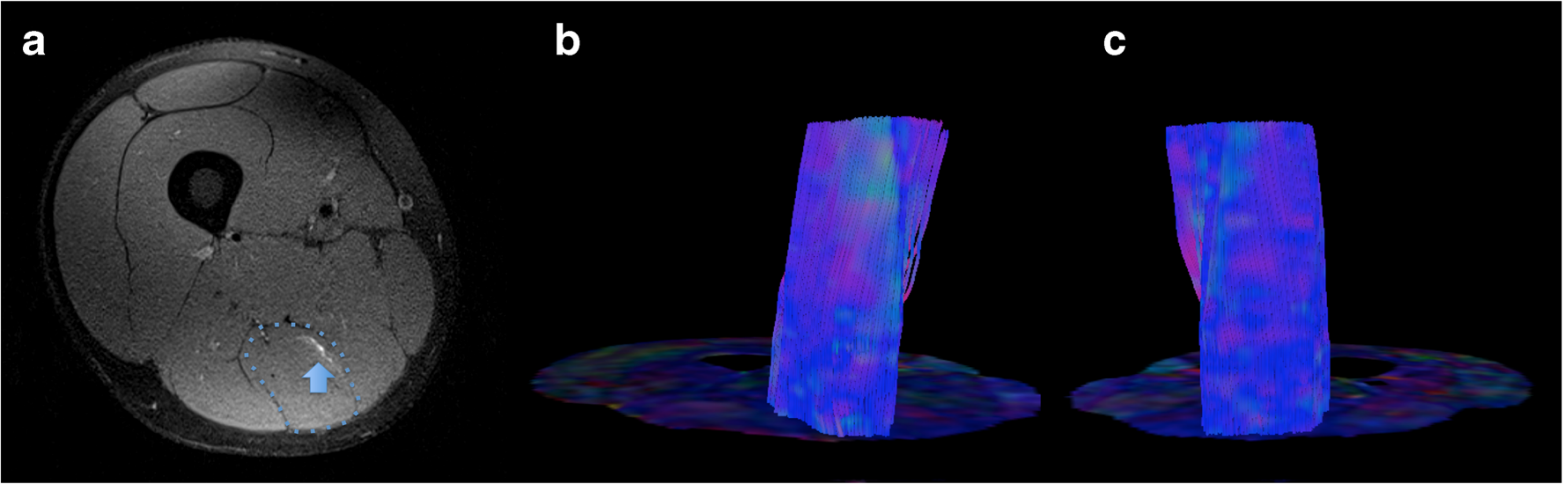
Axial proton density fat-sat image showing a grade I muscle tear of the right semitendinosus muscle (blue arrow in **a**) of a 20-year-old professional football player. In (**b**) and (**c**), the colour-coded maps of the right and left thigh, respectively, are presented along with the corresponding fibre tracking of both semitendinosus muscles (i.e. blue dotted line in **a**), which do not demonstrate any difference for diffusion tensor imaging (DTI) metrics at the statistical analyses (i.e. Student’s t-tests)

The MD, FA, AD and RD values (mean ± SD) of the injured and corresponding contralateral muscles were 1.35 ± 0.10 (×10^-3^ mm^2^/s), 0.20 ± 0.06, 1.73 ± 0.16, 1.16 ± 0.09 and 1.30 ± 0.05 (×10^-3^ mm^2^/s), 0.20 ± 0.05, 1.67 ± 0.12, 1.11 ± 0.05, respectively. The mean ± SD of number, length and volume of the fibre tracts were 8,117 ± 6,348, 44.6 ± 19.2 mm and 93,958 ± 57,292 mm^3^ for the injured muscles, and 8,795 ± 6,402, 46.7 ± 20.9 mm, 108,564 ± 66,799 mm^3^ for the healthy contralateral. No differences emerged for any of the DTI metrics (*p*>0.05, each) (Fig. 2) except for AD (*p*=0.048) (Table 2).

**Table 2:** Entire muscle analyses. Comparison between the injured muscle and the contralateral corresponding healthy muscle

	Entire muscle with tear (mean ± SD)	Entire contralateral healthy muscle (mean ± SD)	Student’s t-test
			*p* value*
tr_n_	8116 ± 6347	8794 ± 6402	0.396
tr_l_ (mm)	44.6 ± 19.16	46.74 ± 20.85	0.496
tr_v_ (mm^3^)	93,957.61 ± 57,291.74	108,564.36 ± 66,799.11	0.189
FA	0.20 ± 0.06	0.20 ± 0.05	0.858
MD (10^-3^mm^2^/s)	1.35 ± 0.10	1.30 ± 0.05	0.078
AD	1.73 ± 0.16	1.67 ± 0.11	**0.048**
RD	1.16 ± 0.09	1.11 ± 0.05	0.106

*tr*_*n*_ number of tracks, *tr*_l_ length of tracks, *tr*_*v*_ volume of tracks, *FA* fractional anisotropy, *MD* mean diffusivity, *AD* axial diffusivity, *RD* radial diffusivity

*Bold type indicates statistically significant values (*p*<0.05)

#### ROI

ROI analyses allowed an improved characterization of muscle injuries as listed in Table 3. The average volume and amount of voxels of the ROIs were 3,942 ± 2,915mm^3^ and 381 ± 282. No differences in DTI metrics were found between ROIs placed in healthy tissue areas (*p*>0.05, each).

**Table 3:** Region of interest (ROI)-based diffusion tensor imaging (DTI) analyses

					1-Way ANOVA *(P)*	*Post-hoc tests^*					
	ROI_tear_	ROI_hc_t_	ROI_hi_	ROI_hc_i_		ROI_tear_ vs. ROI_hc_t_	ROI_tear_ vs. ROI_hi_	ROI_tear_ vs. ROI_hc_i_	ROI_hc_t_ vs. ROI_hi_	ROI_hc_t_ vs. ROI_hc_i_	ROI_hi_ vs. ROI_hc_i_
tr_n_	972 ± 997	1,633 ± 1317	1,002 ± 669	893 ± 661	0.182	-	-	-	-	-	-
tr_l_ (mm)	37.30 ± 22	54.15 ± 24.83	48.08 ± 23.1	44.10 ± 23.7	**0.043**	0.065	0.596	1.000	1.000	0.366	1.000
tr_v_ (mm^3^)	14,530 ± 13,925	22,424 ± 12,960	14,371 ± 10,191	13,482 ± 11,349	0.102	-	-	-	-	-	-
FA	0.18 ± 0.05	0.20 ± 0.04	0.22 ± 0.05	0.20 ± 0.04	**0.008**	**0.018**	**0.015**	0.098	0.454	1.000	1.000
MD (10^-3^mm^2^/s)	1.44 ± 0.11	1.31 ± 0.07	1.33 ± 0.13	1.28 ± 0.1	**0.002**	**0.003**	0.084	**0.002**	1.000	1.000	1.000
AD	1.8 ± 0.15	1.7 ± 0.15	1.7 ± 0.18	1.65 ± 0.17	**0.001**	**0.007**	0.336	**0.007**	1.000	0.784	0.282
RD	1.27 ± 0.12	1.12 ± 0.07	1.14 ± 0.13	1.10 ± 0.08	**0.003**	**0.005**	**0.047**	**0.003**	1.000	1.000	1.000

Comparison between the muscle tear and the healthy contralateral and ipsilateral muscle areas

*tr*_*n*_ number of tracks, *tr*_l_ length of tracks, *tr*_*v*_ volume of tracks, *FA* fractional anisotropy, *MD* mean diffusivity, *AD* axial diffusivity, *RD* radial diffusivity

*ROI*_*tear*_ region of interest drawn on the muscle tear, *ROI*_*hc_t*_ ROI drawn on the on the corresponding healthy contralateral muscle, *ROI*_*hi*_ ROI drawn on a healthy area ipsilateral to the injury, *ROI*_*hc_i*_ ROI drawn on an area matching the ROI_hi_ on the contralateral limb, *^* Greenhouse Geisser

Bold type indicates statistically significant values (*p*<0.05)

The injured areas (i.e. ROI_tear_) showed higher MD (+10.3% than ROI_hc_t_ and +12.3% than ROI_hc_i_, respectively; *p*<0.05 each) and higher AD values (+6.6% than ROI_hc_t_ and +9.1% than ROI_hc_i_, respectively; *p*<0.05, each) than the contralateral healthy areas. There were no differences compared to the ipsilateral healthy regions (i.e. ROI_hi_) (*p*>0.05 for each DTI metric).

**Fig. 3:**
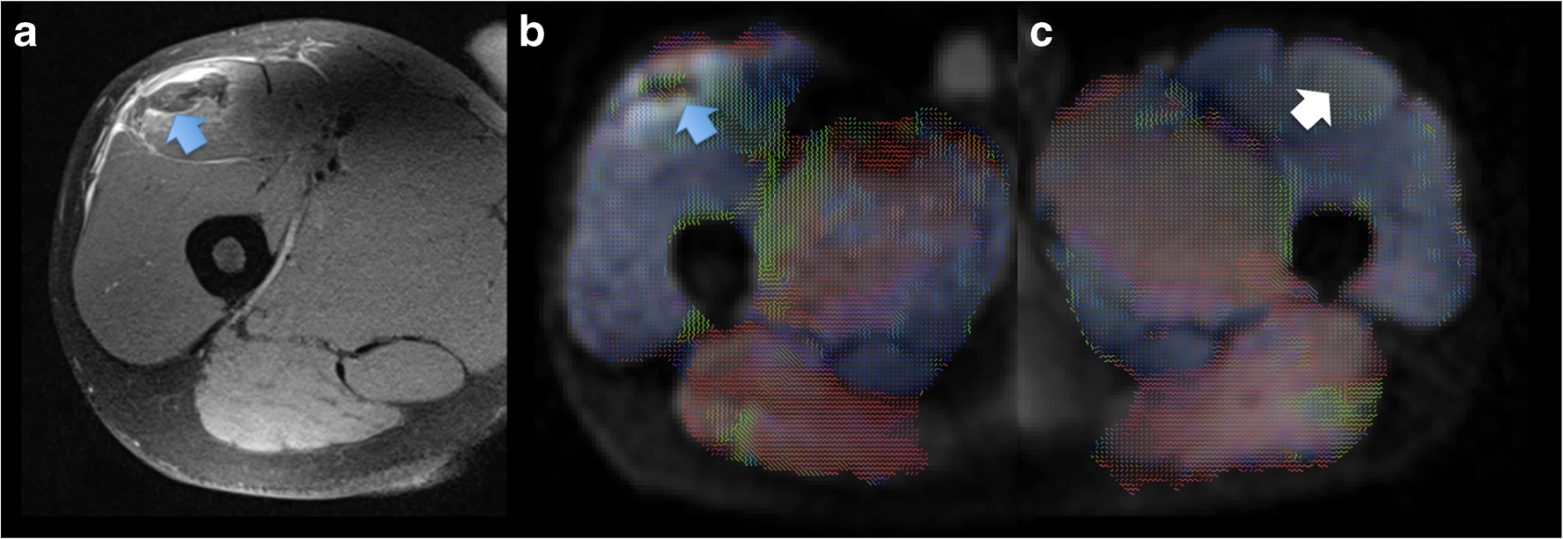
Grade II muscle tear of the right rectus femoris muscle (blue arrow on the axial proton density fat-saturated image in **a**) of a 23-year-old professional football player. The injured area demonstrates lower fractional anisotropy (FA) (blue arrow on the FA map in **b**) than the corresponding healthy contralateral muscle (white arrow on the FA map in **c**)

Also concerning FA, the differences were inhomogeneous. Even if FA was lower in the injured areas (i.e. ROI_tear_) than in the ipsilateral healthy ones (-19.8 % than in ROI_hi_; *p*=0.002) (Fig. 3), differences in the contralateral side emerged only with the healthy ROIs specular to the tear (-11.5 % than in ROI_hc_t_; *p*=0.003). No differences of FA were found between tears (ROI_tear_) and contralateral areas corresponding to the healthy ROI on the injured side (ROI_hc_i_; *p*>0.05).

RD was higher in muscle tears than in any other examined ROIs (+13.1 % than ROI_hc_t_, +10.5 % than ROI_hi_, and +14.8 % than ROI_hc_i_; *p*<0.05).

**Fig. 4:**
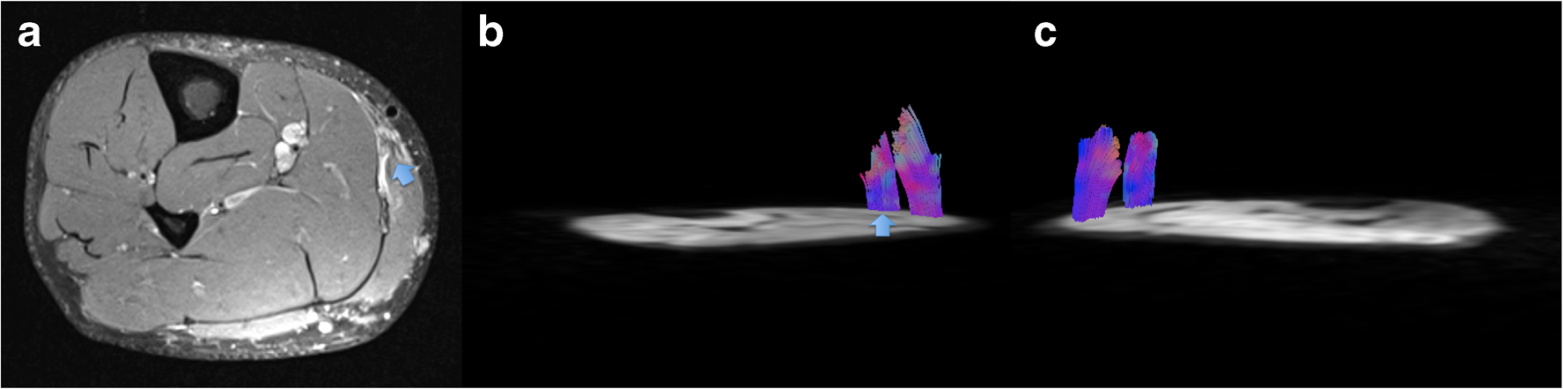
Grade II lesion of the right medial gastrocnemius (blue arrow on the axial proton density fat-saturated image in **a**) of a 35-year-old football player. Fibre tracking of the injured area is illustrated (blue arrow in **b**) and of the ipsi- (**b**) and contralateral healthy areas (**c**). Although visually the fibre tracking of the injured muscle area seems to demonstrate shorter and less numerous fibres, no statistically significant differences occurred in our population comparing the tears with all healthy areas. The statistical analyses revealed significant differences in terms of length and amount of fibre tracts only when a ratio between the ROIs on the injured (i.e. represented here by the fibre tracts on the right calf in **b**) and contralateral extremity (i.e. represented here by the fibre tracts on the left calf in **c**) was calculated

There were no differences for number, length and volume for fibre tracts in any of the performed comparisons (*p*>0.05, each) (Fig. 4).

#### Ratio

The differences between healthy and injured muscles, particularly the fibre-tracking parameters, were more pronounced after normalization (Table 4). Comparison of the ratios (ROI_tear_/ROI_hi_ and ROI_hc_t_/ROI_hc_i_) revealed higher MD and RD (+6 % and +8.7 %, respectively; *p*<0.05 each) and lower FA (-19.5 %, *p*=0.07) as well as a reduced number and length of fibre tracts on the injured side (-55.6 % and -39.5 %, respectively; *p*<0.05) (Fig. 4). There were no differences for AD and fibre tract volume (*p*>0.05, each).

**Table 4:** Comparison of diffusion tensor imaging (DTI) metrics’ ratio between the injured leg and the contralateral healthy one

	ROI_tear_/ROI_hi_	ROI_hc_t_/ROI_hc_i_	Students’ t-test
tr_n_	0.55 ± 0.45	1.24 ± 0.53	**0.028**
tr_l_ (mm)	0.69 ± 0.22	1.14 ± 0.28	**0.005**
tr_v_ (mm^3^)	0.62 ± 0.40	1.26 ± 0.65	0.056
FA	0.88 ± 0.07	1.09 ± 0.15	**0.007**
MD (10^-3^mm^2^/s)	1.10 ± 0.04	1.04 ± 0.07	**0.028**
AD	1.06 ± 0.03	1.04 ± 0.04	0.241
RD	1.13 ± 0.06	1.03 ± 0.10	**0.014**

*tr*_*n*_ number of tracks, *tr*_l_ length of tracks, *tr*_*v*_ volume of tracks, *FA* fractional anisotropy, *MD* mean diffusivity, *AD* axial diffusivity, *RD* radial diffusivity, *ROI*_*tear*_*/ROI*_*hi*_ ratio between the ROI drawn on the tear and the one drawn on a ipsilateral healthy area, *ROI*_*hc_t*_*/ROI*_*hc_i*_ ratio of the two corresponding contralateral healthy areas

Bold type indicates statistically significant values (*p*<0.05)

## Discussion

Our results suggest that normalized DTI/fibre-tracking metrics obtained via artifact-corrected STEAM-DTI are insensitive to possible bias due to laterality, being thus well suited for quantitative diagnostic assessment of muscle tears.

Acute muscle tears are characterized by alterations of the myofibrillar structure and inflammation [39, 40]. DTI is uniquely sensitive to changes in the magnitude and directionality of intramuscular water diffusivity occurring in acute muscle tears. Hence, these alterations are expected to be easily detected and quantified by this technique. Our results show an absence of significant differences (i.e. besides higher AD on the injured side) comparing entire and injured muscles. This is consistent with observations by McMillan et al. on an animal model for injuries of the tibialis anterior [13]. Indeed, these authors found significant differences in DTI metrics only comparing wild and dystrophic mice with muscle injury or comparing injured and non-injured dystrophic animals, whereas differences between injured and non-injured wild animals did not occur [13].

In contrast, Zaraiskaya et al. [14] showed significant differences in FA, MD and eigenvalues (i.e. λ_1_,λ_2_,λ_3_) comparing DTI measures from entire healthy muscles (i.e. eight volunteers) with those obtained in ROIs drawn in injured muscle areas of the calves of four patients (i.e. two with haematomas and two with muscle tears). These results are in accordance with the differences in FA, MD, RD and AD found in our population comparing the injured areas with the healthy ones (i.e. ipsi- and contralateral ROIs), even if it has to be taken into account that the presence of oedema may lead just to an apparent decrease of AD and FA [41].

Zaraiskaya et al. performed fibre tracking only in healthy controls, but no such data were presented for patients [14]. Froeling et al. [35] evaluated fibre-tracking changes at different time points in marathon runners, but performed no separate assessments for muscle strains already visible on anatomical images (i.e. T2w images). To the best of our knowledge, there are no previous studies that have investigated fibre-tracking metrics (i.e. number, length and volume of tracked fibres) of muscle tears. In our cohort, no differences in fibre tracking emerged, either in the entire muscle, or in the ROI-based analyses. The tracked muscle fibres of the injured side turned out to be significantly less numerous (-55 %) and shorter (-39 %) only after normalization of the data. Considering that in athletes an asymmetry in the characteristics and metabolic activity of muscle belonging to the dominant and non-dominant side has been shown [30–33], it appears reasonable that the laterality is a biasing factor in quantitative DTI assessments in muscles. The results obtained after applying the normalization seem to confirm this assumption, as differences in length, number and volume of the tracked fibres due to the injury were apparent only in normalized data.

Our study results are preliminary and could not yet validate the fact that STEAM-DTI brings any additional benefits compared to conventional MRI. Since one of the more challenging aspects of muscle tear assessment is represented by the prognosis of the recovery interval [42], we strongly believe that the application of the ratio could also provide essential benefits for the longitudinal evaluation of muscle strains during the recovery phase and thus improve the prediction of the recovery interval and reduce the risk of recurrence.

## Limitations

Despite our very promising results, there are some limitations to our study. All patients were scanned within 1 week after the injury; however, DTI/fibre-tracking metrics may change quite quickly (e.g. inflammation may occur in a few days). Thus, a more standardized recruitment (i.e. a fixed number of days after the injury for all patients) may be beneficial, especially for entire muscle analyses. Despite the evidence that in volunteers different ranges of DTI metrics values occur in different muscles [15, 16], in the present study separate analyses according to the injured muscles were not performed, because of the low number of examined patients. Future studies including larger patient populations should focus on muscle-specific analyses to provide even more accurate results. Nevertheless, normalization will certainly also reduce such differences between muscle groups.

Finally, the quite long acquisition time (i.e. ca. 8 min) might represent a limit with very extensive lesions, since motion artifacts may occur. However, recent developments in simultaneous-multi-slice (SMS) DTI have translated into ~threefold acceleration of clinically available DTI sequences [43]. SMS was not yet implemented into our STEAM-DTI sequence when our study was performed, but future studies aiming for larger FOVs should directly benefit from this new technology.

## Conclusion

In conclusion, STEAM-based DTI allowed a precise assessment of the injured fibres in athletes especially when a ratio between the injured and the contralateral muscles was applied. Aiming to improve the current imaging-based classification of muscle tears and to increase the accuracy of the therapeutic and prognostic management of injured athletes, future studies including a larger population and evaluating muscle tears, also during follow-up, are necessary.

## Abbreviations

ROI_tear_: Region of interest (ROI) drawn on the muscle tear
ROI_hc_t_: ROI drawn on the corresponding healthy contralateral muscle
ROI_hi_: ROI drawn on a healthy area ipsilateral to the injury
ROI_hc_i_: ROI drawn on an area matching with ROI_hi_ on the contralateral limb
